# Effect of Root Canal Dressings on Extraradicular pH in a Sealed Apex Model: **An In Vitro Study**

**DOI:** 10.1002/cre2.70467

**Published:** 2026-09-27

**Authors:** Alina Paganini, Eva Magni, Wadim Leontiev, Thomas Connert, Anne Géraldine Guex, Andreas Filippi

**Affiliations:** ^1^ Department of Oral Surgery University Center for Dental Medicine Basel UZB University of Basel Basel Switzerland; ^2^ Center of Dental Traumatology, University Center for Dental Medicine Basel UZB University of Basel Basel Switzerland; ^3^ Department of Periodontology Endodontology and Cariology, University Center for Dental Medicine Basel UZB University of Basel Basel Switzerland; ^4^ Department Research University Center for Dental Medicine Basel UZB University of Basel Basel Switzerland; ^5^ Department of Biomedicine University of Basel Basel Switzerland

**Keywords:** calcium hydroxide, dental trauma, Ledermix, root resorption

## Abstract

**Objectives:**

Endodontic treatment is mandatory after severe dental trauma such as avulsion or intrusion. Initial intracanal dressing with calcium hydroxide should be avoided according to international guidelines due to potential periodontal ligament damage by pH elevation. However, whether an intracanal dressing is able to alter the extraradicular site remains unclear. This in vitro study quantified extraradicular pH changes through dentinal diffusion of calcium hydroxide or Ledermix in a sealed‐apex model in human teeth.

**Material and Methods:**

Thirty‐five cryopreserved human premolars were prepared with or without cemental defects and received UltraCal XS, Ledermix, or saliva as an intracanal dressing. In the test groups, apices were sealed with composite resin material; the positive control remained with open apices. Teeth were stored in NaCl solution. External pH of the solution was measured on days 1, 3, 5, 7, and 14. A mixed model was applied to determine the effect of group, apical plug, cemental defect, and time on pH alterations.

**Results:**

Calcium hydroxide in teeth without apical sealing caused a sustained external alkalinisation (± pH 12). In teeth with apical sealing, no significant external rise in pH occurred for any dressing over time compared to day 1; only one sealed specimen with calcium hydroxide dressing reached pH 9.1 transiently. The pH in Ledermix and saliva groups remained roughly neutral irrespective of sealing or cemental defects. Mixed‐effects modeling identified the apical plug as the only significant factor (*p* < 0.05); presence of cemental defects or days after treatment had no significant effect on pH.

**Conclusions:**

In a sealed‐apex model, intracanal dressings did not raise the external pH significantly by dentinal diffusion. Therefore, the results do not support the advantage of a certain intracanal dressing in endodontic treatment after severe dislocation injuries of teeth without open apices with respect to maintained physiological pH.

## 1 Introduction

Severe dislocations such as avulsion and intrusion are the most severe forms of dental trauma. These injuries are frequently associated with poor long‐term prognosis due to the risk of root resorption and ankylosis (Fouad et al. 2020). The survival of the periodontal ligament (PDL) cells on the root surface at the time of reposition or replantation is the most decisive factor for periodontal healing (Bryson et al. 2002; Day et al. 2012). When these cells are severely damaged, the risk of replacement resorption and ankylosis increases significantly (Wong and Sae‐Lim 2002; Barbizam et al. 2015). For avulsed or dislocated teeth > 1 mm, with fully formed apices, root canal treatment is considered mandatory (Krastl et al. 2021; Trope et al. 1992). Without timely extirpation of the pulp, bacterial degradation products and toxins may diffuse through dentinal tubules and the apical foramen, initiating inflammatory responses in the surrounding periodontal tissues, ultimately leading to infection‐related root resorptions (Stewart et al. 2008). Therefore, endodontic disinfection and the choice of intracanal medication are of particular importance for the prognosis after severe dislocation injuries (Pierce and Lindskog 1987).

The objective of intracanal dressings in dental traumatology differs fundamentally from the use in caries‐related endodontic infections: The aim is not only to disinfect the root canal system, but rather to prevent post‐traumatic microbial contamination and subsequent infection‐related or replacement resorption (Andreasen et al. 1995, 2013). Current international literature, such as from the International Association for Endodontology, recommends either delayed initiation of root canal treatment with calcium hydroxide after 7–10 days and maintaining it for 2 weeks before root canal filling in mature teeth, or rather the immediate placement of corticosteroid–antibiotic dressings in avulsed or severely dislocated teeth (Krastl et al. 2021). This recommendation is based on earlier studies demonstrating that corticosteroid dressings reduce replacement resorption by suppressing the inflammatory response in the periodontal ligament (Thong et al. 2001; Chen et al. 2008; Kirakozova et al. 2009).

The most widely used dressing, Ledermix Paste, contains triamcinolone acetonide and demeclocycline hydrochloride. While triamcinolone provides anti‐inflammatory effects (Pierce et al. 1988), demeclocycline is widely antimicrobial. As a tetracycline, demeclocycline may cause crown discoloration (Chen et al. 2014). It is known that tetracycline is not required, as corticosteroids alone produce the same effect as the combined medication (Trope 2011), which is why alternative corticosteroid‐products without antibiotics have been introduced (Kendell‐Wall et al. 2024). However, commercial availability remains limited, justifying the wide use of Ledermix. On the other hand, calcium hydroxide is considered the standard intracanal compound in endodontic treatment because of its remarkable antibacterial activity (Jana et al. 2025). The antibacterial mechanism is based on the release of hydroxide (OH^−^), creating an alkaline environment (pH ≈12), which then again also exerts cytotoxic effects on periodontal and cementoblast‐like cells (Cai et al. 2018). Since mammalian cells begin to lose viability above pH 7.8 (Lengheden 1994), diffusion of hydroxide through dentinal tubules or apical openings may cause additional periodontal damage after trauma. Although hydroxide diffusion through dentine has been demonstrated with higher pH in inner and outer dentin after calcium hydroxide‐dressings (Nerwich et al. 1993; Tronstad et al. 1981), it remains unclear whether such diffusion can alter the pH on the extraradicular site (Zare Jahromi and Kalantar Motamedi 2019). Investigating this will significantly deepen our understanding of the broader influence of distinct root canal dressings with respect to the surrounding tissue and allow for evidence‐based, informed decisions in clinical settings. The present in vitro study aimed to compare the extraradicular pH alterations in human teeth dressed with UltraCal XS (a widely used calcium hydroxide paste) or Ledermix Paste under standardized laboratory conditions. The study examined solely the effect of ion diffusion through dentinal tubules on external pH changes. The hypothesis was that UltraCal XS would result in a significant increase in extraradicular pH despite apical sealing. Furthermore, pH changes were expected to be greater in teeth with cemental defects compared with teeth without defects.

## 2 Materials and Methods

The manuscript of this laboratory study has been written according to Preferred Reporting Items for Laboratory studies in Endodontology (PRILE) 2021 guidelines (Nagendrababu et al. 2021). The completed PRILE checklist and flowchart are provided as Supporting Information S1: Files 1 and 2, respectively.

### 2.1 Thawing of the Cryopreserved Teeth

This study was performed on teeth that were extracted based on clinical indication not related to this study. The extracted teeth were cryopreserved between 2008 and 2019 after written informed consent was obtained from all participants or their parents. Their use was approved by the research ethics committee. The teeth were irreversibly anonymized and randomly allocated to the study groups by A.P. and E.M. Thirty‐five non‐carious and intact, one‐rooted, cryopreserved human premolars were chosen for this in vitro study. To thaw the teeth, storage containers were removed from a liquid nitrogen tank and transported on dry ice at –78.5°C. Thawing was performed in a 37°C water bath with gentle agitation of the container. Once the medium–cryoprotectant mixture was liquefied, the teeth were removed and rinsed three times in Petri dishes containing PBS. Because the study aimed to simulate avulsed teeth, they were not stored in a medium for keeping the cells vital, but in a tube with sodium chloride (NaCl 0.9% Isotonic Sodium Chloride Irrigation Solution, OMNIA S.r.l., Fidenza, Italy).

### 2.2 Preparation of Specimens

**Figure 1:**
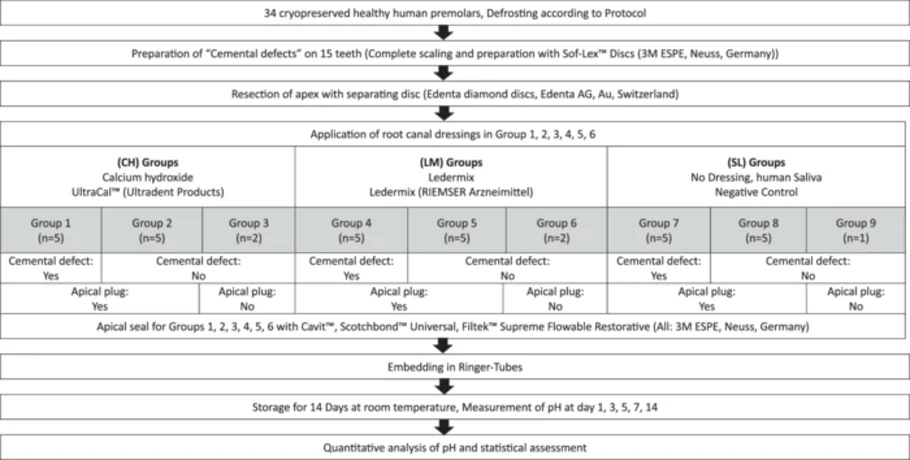
Study protocol: Schematic overview of the experimental workflow using 34 cryopreserved healthy human premolars. Following standardized defrosting, cemental defects were prepared, the apices were resected, and different intracanal dressings (calcium hydroxide, Ledermix, or human saliva) were applied in defined experimental groups with or without an apical plug. All specimens in Groups 1–6 received an apical seal and were embedded in Ringer tubes. Samples were stored at room temperature for 14 days, with pH measurements performed on days 1, 3, 5, 7, and 14, followed by quantitative and statistical analysis.

**Figure 2:**
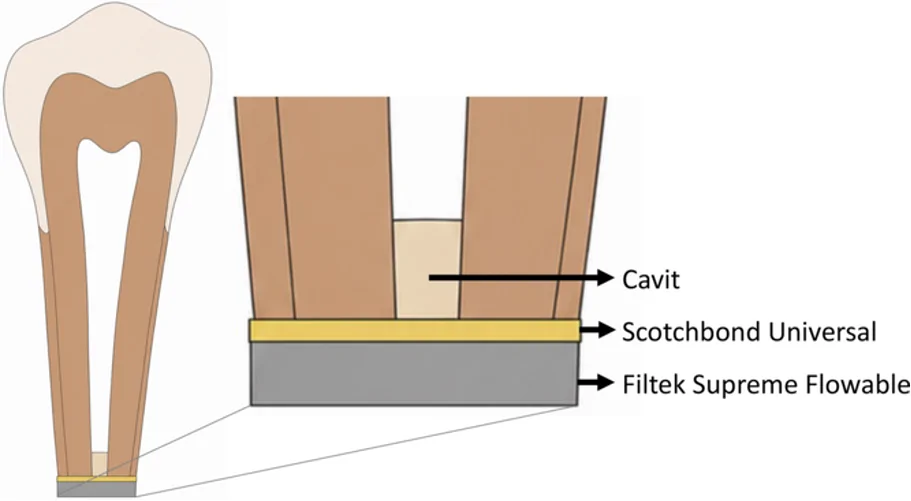
Apical sealing procedure: Schematic illustration of the apical seal used to close the root apex following root canal preparation. A 2–3 mm layer of Cavit was placed within the apical canal space, followed by application of Scotchbond Universal adhesive in self‐etch mode and coverage with Filtek Supreme Flowable Restorative to seal the apical surface.

Twenty teeth were left with an intact root surface, while 15 teeth had the periodontal cells completely removed using scalers and Sof‐LexDiscs (3 M ESPE, Neuss, Germany) and were then rinsed thoroughly with NaCl solution. Subsequently, all apices were resected (2 mm) using a sharp separating disc (Edenta diamond discs, Edenta AG, Au, Switzerland). The pulp tissue of the teeth in the Calcium hydroxide (CH) and Ledermix (LM) groups (1–6) was then removed retrogradely with a pulp extirpator (VDW STERILE Barbed Broaches ISO XXF, VDW GmbH, Munich, Germany) and RECIPROC R25 files (VDW GmbH, Munich, Germany) with a dedicated reciprocating endodontic motor (VDW. Gold, RECIPROC, VDW GmbH, Munich, Germany), while the canals were repeatedly irrigated with NaCl solution. To prevent dehydration, teeth were continuously wrapped in a gauze soaked with NaCl. An overview of study groups is shown in Figure 1. In the CH groups, UltraCal XS (Ultradent Products Inc., South Jordan, UT, USA) dressings were placed with high precision tips (CALASEPT Plus Flexi‐needles, Directa AB, Upplands Väsby, Sweden); however, only in groups 1 and 2 the apex was sealed using Cavit (2–3 mm layer) (3M ESPE, Neuss, Germany), Scotchbond Universal (3M ESPE, Neuss, Germany) and Filtek Supreme Flowable Restorative (3M ESPE, Neuss, Germany). The bonding agent was applied in self‐etch mode according to the manufacturer's instructions, rubbed in for 20 s, gently air‐dried for 5 s, and light‐cured for 10 s at 1200 mW/cm^2^ using an LED curing unit (Bluephase G2, Ivoclar Vivadent, Schaan, Liechtenstein). The apical seal is illustrated in Figure 2. Group 3 served as a positive control without an apical plug. Similarly, in the LM groups, Ledermix dressings (RIEMSER Arzneimittel GmbH, Greifswald, Germany) were placed, with groups 4 and 5 receiving apical plugs, while group 6 served as a positive control without apical sealing. The composition of the used dressings and the original pH are listed in Table 1. The saliva (SL) groups did not receive any intracanal dressing; instead, they were intracanally rinsed with human saliva to simulate the bacterial load present in the oral cavity. The saliva was pooled from five healthy individuals at 12 pm, without prior food or drink intake for 4 h. Groups 7 and 8 were then apically sealed following the same procedure as described, group 9 was left open. The SL groups served as negative controls to assess whether the growth of microorganisms would influence the pH value. All teeth were stored in 5 mL Eppendorf safe‐lock tubes embedded in 1 mL of NaCl solution.

**Table 1:** Overview of the origin, composition, and pH of the intracanal dressings used in this study.

Product	Manufacturer	Composition	Reported pH
UltraCal XS	Ultradent Products Inc., South Jordan, Utah, USA	Calcium hydroxide (Ca(OH)_2_), barium sulfate, aqueous suspension medium	12.0–12.5
Ledermix Paste	RIEMSER Arzneimittel GmbH, Greifswald, Germany	Demeclocycline hydrochloride (3.2 mg/g), triamcinolone acetonide (1 mg/g), polyethylene glycol base	7.8–8.2

### 2.3 Measurement of pH

The pH value was measured after 1, 3, 5, 7, and 14 days. The tubes were stored at room temperature and briefly opened for each measurement. The pH electrode (SevenExcellence pH meter S400, Mettler‐Toledo GmbH, Greifensee, Schweiz) was inserted into the NaCl solution to record the pH value. The electrode was thoroughly cleaned between each measurement, and the same procedure was repeated for all teeth. The electrode was recalibrated daily, and the measurements were randomly cross‐checked by two different investigators, each performing three measurements to ensure consistency and reliability of the data.

### 2.4 Statistical Assessment

As no pilot data were available for this in vitro study design, a formal a priori power analysis was not feasible. The sample size was determined based on the availability of one‐rooted extracted human premolars and was consistent with previous in vitro studies investigating pH changes associated with calcium hydroxide‐based intracanal medicaments, which used comparable sample sizes (28–41 teeth) (Calt et al. 1999; Wong et al. 2021).

Statistical analysis was performed using a mixed‐effects model to evaluate the influence of the categorical variables Group (type of intracanal dressing: UltraCal Ledermix, saliva), Cemental Defect (yes/no), Apical Plug (yes/no), and Day (1, 3, 5, 7, 14) on the pH values. The model included these variables as fixed effects and accounted for random variability across the different groups to correct for repeated measurements within the same experimental unit. Statistical significance was set at *α* = 0.05. Analyses were conducted in Python (version 3.11) using the statsmodels, pandas, numpy, and seaborn packages.

## 3 Results

### 3.1 pH Changes Over Time

**Figure 3:**
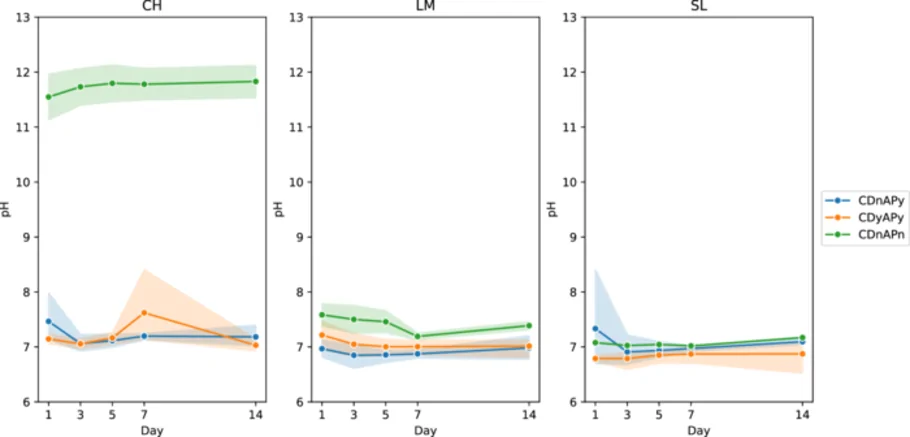
Mean pH values (± 95% confidence intervals) measured after 1, 3, 5, 7, and 14 days for the three groups: Calcium hydroxide (CH), Ledermix (LM), and saliva (SL). Each color represents a subgroup based on the presence or absence of cemental defects (CD) and apical plug (AP): 1 CDnAPy = Cemental Defect: no, Apical Plug: yes (blue). 2 CDyAPy = Cemental Defect: yes, Apical Plug: yes (orange). 2 CDnAPn = Cemental Defect: no, Apical Plug: no (green).

The pH measurements over 14 days showed distinct trends among the CH, LM, and SL groups (Figure 3 and Tables 2 and 3). In the CH groups, specimens without cement defect and without an apical plug (CDnAPn) exhibited a consistently high alkaline pH, stabilizing ±12 throughout the observation period. In contrast, teeth with an apical plug (CDnAPy and CDyAPy) lead to substantially lower pH values, remaining close to neutrality (pH ≈ 6.5–7.5) with minor fluctuations, regardless of the existence of cement defects. Notably, one specimen in the CH group with an apical seal exceeded the critical threshold of pH 7.8, resulting in a maximum value of 9.1 on day 7 (Table 2). In the LM and SL groups, the pH values remained near neutral across all time points, with slight decreases at day 3 and gradual stabilization thereafter. Mixed‐effects model confirmed these observations statistically:

**Table 2:** Measured pH values for all specimens (#) across the nine experimental groups with calcium hydroxide (CH), Ledermix (LM), or saliva (SL), with mean ± standard deviation (SD) after 1, 3, 5, 7, and 14 days.

Group	#	Cemental defect	Apical plug	Day 1	Day 3	Day 5	Day 7	Day 14
1 (CH)	1	Yes	Yes	7.3	7.0	7.3	9.1	7.2
	2			7.1	6.9	6.9	7.0	6.9
	3			7.3	7.1	7.1	7.3	6.9
	4			7.0	7.2	7.3	7.3	7.2
	5			7.1	7.2	7.2	7.3	7.1
**Mean ± SD**				**7.6** ± **0.1**	**7.1** ± **0.12**	**7.2** ± **0.6**	**7.6** ± **0.9**	**7.0** ± **0.1**
2 (CH)	6	No	Yes	7.0	6.9	6.9	7.1	7.0
	7			7.4	7.2	7.2	7.3	7.3
	8			7.3	7.0	7.1	7.2	7.1
	9			8.5	7.3	7.3	7.3	7.0
	10			7.1	6.9	7.0	7.2	7.6
**Mean ± SD**				**7.5** ± **0.6**	**7.1** ± **0.2**	**7.1** ± **0.2**	**7.2** ± **0.1**	**7.2** ± **0.2**
3 (CH)	31	No	No	12.0	12.0	12.1	12.1	12.1
	32			11.1	11.4	11.5	11.5	11.5
**Mean ± SD**				**11.6** ± **0.6**	**11.7** ± **0.5**	**11.8** ± **0.5**	**11.8** ± **0.4**	**11.8** ± **0.4**
4 (LM)	11	Yes	Yes	7.6	7.1	7.0	7.2	7.1
	12			7.4	7.4	7.2	7.1	7.1
	13			7.0	7.0	7.0	7.0	7.1
	14			7.2	7.2	7.1	7.0	7.1
	15			6.8	6.7	6.7	6.7	6.6
**Mean ± SD**				**7.2** ± **0.3**	**7.1** ± **0.3**	**7.0** ± **0.2**	**7.0** ± **0.2**	**7.0** ± **0.2**
5 (LM)	16	No	Yes	6.7	6.4	6.6	6.7	6.6
	17			7.1	6.9	6.9	7.0	6.9
	18			7.2	7.1	6.9	6.8	7.4
	19			6.9	7.0	6.9	6.9	7.0
	20			6.9	6.8	7.0	7.0	7.0
**Mean ± SD**				**7.0** ± **0.2**	**6.9** ± **0.3**	**6.9** ± **0.2**	**6.9** ± **0.1**	**7.0** ± **0.3**
6 (LM)	33	No	No	7.8	7.8	7.6	7.1	7.5
	34			7.4	7.3	7.3	7.3	7.3
**Mean ± SD**				**7.6** ± **0.3**	**7.5** ± **0.4**	**7.5** ± **0.3**	**7.2** ± **0.1**	**7.4** ± **0.1**
7 (SL)	21	Yes	Yes	6.9	6.8	6.9	7.0	7.1
	22			6.7	6.9	6.9	7.0	7.1
	23			6.7	6.4	6.6	6.6	6.2
	24			6.8	6.9	7.0	7.1	7.0
	25			6.8	6.9	6.9	6.9	7.0
**Mean ± SD**				**6.8** ± **0.1**	**6.8** ± **0.2**	**6.9** ± **0.2**	**6.9** ± **0.2**	**6.9** ± **0.4**
8 (SL)	26	No	Yes	6.7	6.8	6.9	6.9	7.0
	27			6.8	6.7	6.9	7.0	7.1
	28			6.6	6.6	6.7	6.9	7.1
	29			7.1	7.0	7.0	7.0	7.1
	30			9.4	7.5	7.2	7.1	7.2
**Mean ± SD**				**7.3** ± **1.2**	**6.9** ± **0.6**	**6.9** ± **0.2**	**7.0** ± **0.1**	**7.1** ± **0.1**
9 (SL)	35	No	No	7.1	7.0	7.0	7.0	7.2

**Table 3:** Results of the linear mixed‐effects model evaluating the effects of intracanal dressing group, cemental defect, apical plug, and measurement day on pH values. Regression coefficients (β), standard errors (SE), 95% confidence intervals (CI), and *p*‐values are presented.

Predictor	Estimate	95% CI	*p* value
Group (LM vs. CH)	−0.90	−3.12 to 1.35	0.43
Group (SL vs. CH)	−0.86	−3.05 to 1.34	0.45
Cemental defect	−0.02	−0.28 to 0.24	0.88
Apical plug	−1.97	−2.34 to −1.60	< 0.001
Day 3 vs. Day 1	−0.17	−0.55 to 0.21	0.39
Day 5 vs. Day 1	−0.14	−0.52 to 0.24	0.48
Day 7 vs. Day 1	−0.07	−0.44 to 0.32	0.74
Day 14 vs. Day 1	−0.10	−0.48 to 0.28	0.61

Only the presence of an apical plug significantly reduced the pH value (*p* < 0.05), while the factors group, cemental defect, and time (days 1–14) did not lead to any statistically significant effects (*p* > 0.05) (Table 3). Approximately 50% of the total variance in pH was attributed to random effects, reflecting both inter‐ and intragroup variability not explained by the fixed factors.

### 3.2 Effect of Cemental Defects

The presence or absence of cemental defects did not influence extraradicular pH in any experimental group. No statistically significant differences were observed between teeth with intact cementum and those with mechanically created cemental defects (*p* > 0.05) (Figure 3 and Table 2).

## 4 Discussion

This in vitro study aimed to determine extraradicular pH changes due to dentinal diffusion of two different root canal dressings, calcium hydroxide or Ledermix, respectively, in a sealed‐apex model. The results demonstrated that there was no significant alteration of the pH value for both dressings in the closed apex model. However, the absence of an apical plug led to an increase of the pH in the calcium hydroxide group. Within the conceptual boundaries of this model, these data suggest that dentinal tubule diffusion of root canal dressings alone may be more limited than previously assumed. The first hypothesis, that extraradicular pH would rise after calcium hydroxide dressing solely through dentinal diffusion, therefore was rejected. No statistically significant differences were observed between teeth with intact cementum and those with mechanically created cemental defects, which supports the rejection of the hypotheses that UltraCal XS would increase extraradicular pH despite sealing and that teeth with cemental defects would show greater pH changes.

This question is clinically relevant because to date, there is no clear consensus on whether calcium hydroxide can or should be placed in severely dislocated teeth immediately after trauma. The International Association for Dental Traumatology (IADT) recommends either the immediate placement of a corticosteroid dressing or the use of calcium hydroxide within the first 2 weeks, without further consideration of early pulp infection and the subsequent risk of infection‐related root resorption (Fouad et al. 2020). The International Association for Endodontology recommends the early/immediate initiation of root canal treatment and initial use of corticosteroid–antibiotic dressings rather than calcium hydroxide, but discusses also limited data on a clear recommendation (Krastl et al. 2021). The most widely known corticosteroid paste, Ledermix, has a pH of approximately 8, slightly above the toxic threshold of 7.8, and is therefore not expected to significantly increase extraradicular pH.

According to published literature, calcium hydroxide is not recommended for immediate use due to its toxicity and irritant properties (Abbott 2016), a position that is primarily grounded in earlier‐generation studies from 1981 to 1991 (Nerwich et al. 1993; Tronstad et al. 1981; Lengheden et al. 1991; Lengheden and Blomlöf 1991). Early studies reported rises in inner and outer dentine pH after calcium hydroxide dressings, but not in the cementum, which may serve as a barrier (Plataniotis and Abbott 1999; Teixeira et al. 2005). These findings align with Calt et al. (1999) who also observed in a sealed model with cemental defects that even when Ca^2+^ and OH^−^ ions were detectable, pH values did not rise. These results are in line with the findings in the present study. Also, Fuss et al. (1989) compared pH changes in a sealed model of tubular diffusion for bleaching agents and calcium hydroxide, and found that bleaching agents could diffuse and alter periradicular pH, whereas no pH increase was detectable with calcium hydroxide. Esberard et al. (1996a) monitored pH changes at the cemental surface for up to 120 days after root filling with calcium‑hydroxide‑based sealers and found no increase. The same group subsequently drilled perforations in roots and placed calcium hydroxide dressings, after which they could detect an increase in pH over time (Esberard et al. 1996b). In contrast to the present findings, Guerreiro‐Tanomaru et al. (2012) detected a pH rise in a sealed model (peaking at 8.29 after 21 days).

This newly developed in vitro model demonstrated reliable results on this question, as the control groups neither exhibited pH decreases due to microbial acidification over time nor showed any influence of the composite material itself on pH. Resin‐based composites are known not to exhibit buffering capacity and to maintain a stable pH over an observation period of up to 15 days (Lehmann et al. 2021; Nedeljkovic et al. 2016). The use of cryopreserved human teeth is supported by prior evidence showing preservation of PDL vitality and absence of relevant microcracks (Oh et al. 2005; Kühl et al. 2012). Specimens were stored in NaCl solution and not maintained in optimized cell culture media or conditions, which will lead to gradual cell deterioration. In one experimental group, the cementum was completely removed, and in the other it was left on the teeth surface, potentially deviating into a necrotic state but still serving as a potential barrier. The effect of this barrier on extraradicular pH changes could not be demonstrated, therefore, the second hypothesis was also rejected. Storage in NaCl solution was inevitable since storage in a cell‐preserving medium would first have led to the neutralization of possible pH changes, since all cell‐preserving substances possess buffering capacities. Second, a cell‐preserving medium would have provided more favorable conditions for bacterial growth, which again might have falsified the pH measurements or would have necessitated experimental work under strictly aseptic conditions. Considering that the realistic clinical scenario following traumatic dental injuries primarily involves removal of pulp tissue rather than conventional mechanical root canal preparation, pulp extirpation was performed using an extirpator, with rotary instruments used only to assist tissue removal. Therefore, extensive mechanical instrumentation and subsequent irrigation protocols aimed at smear layer removal were not performed. NaOCl was intentionally avoided because its alkaline properties could influence the measured pH values and introduce an additional variable unrelated to the specific research question. A limitation of this study, however, is that NaOCl may have affected smear layer removal and dentinal tubule permeability, and the findings should be interpreted within the context of this experimental model. Additionally, in vivo, replanted teeth are surrounded by blood and subsequently by periodontal and bone tissue, all of which act as chemical buffers. In vivo, such buffering would be expected to neutralize, whereas NaCl has poor buffer capacities as such. Therefore, the pH changes observed in the present in vitro model should be interpreted as physicochemical findings only and should not be directly extrapolated to the in vivo situation, where these physiological conditions may substantially modify the local environment. An additional limitation relates to the characterization of the included teeth, which was based on visual inspection only. No three‐dimensional imaging method, such as micro‐computed tomography, was performed to identify potential anatomical variations, microcracks, accessory canals, or other factors that may influence calcium hydroxide diffusion. Although micro‐computed tomography would provide a more comprehensive evaluation, its application within the present experimental design was limited due to potential artifacts associated with scanning after experimental procedures and apical sealing. A further limitation is that only two apical configurations (open/sealed) were tested, it remains plausible that the extent of external pH elevation is dependent on the diameter of the apical construction. Studies have shown larger diameters leading to a higher OH^−^ release (Wong et al. 2021; Robert et al. 2005). Teeth with wide‐open apices therefore might be at bigger risk for additional cemental damage due to pH alterations by calcium hydroxide compared to teeth with fully formed apices and a small diameter of the apical foramen. Also, the thickness of the dentinal wall as well as the degree of dentinal tubules obliteration might influence the diffusion rate of ions and therefore might have an influence on pH alterations. The isolated increase in pH to 9.1 in one specimen with an apical seal on day 7 is consistent with the expected time‐dependent diffusion pattern of calcium hydroxide (Sharma et al. 2018), with a pronounced effect observed after approximately 1 week. However, specimen‐specific factors, such as anatomical variations (e.g., accessory canals) or differences in dentinal permeability related to dentinal wall thickness, may have contributed to this individual finding. As the reported antimicrobial effectiveness of calcium hydroxide beyond 7 days remains inconsistent, a 14‐day observation period was considered clinically relevant and was therefore selected for the present study (Sharma et al. 2018).

UltraCal XS is a commercially available premixed calcium hydroxide paste intended for intracanal medication. The convenient application has made premixed calcium hydroxide formulations widely used in daily endodontic practice. Compared with freshly mixed calcium hydroxide, these formulations may contain additional components (Table 1), however, the pH value remains the same.

The only study that has ever used an animal model to compare calcium hydroxide versus Ledermix as an intracanal dressing is a study by Bryson et al. (2002): In six dogs, 15 two‐rooted premolars were surgically separated and extracted, stored dry extra‐orally for 60 min, after which 15 canals were filled with calcium hydroxide and 14 with Ledermix. After 4 months the dogs were euthanized, and the teeth together with the surrounding bone were sectioned and examined histologically. Healing was classified as favorable (presence of cementum) or unfavorable (absence of cementum), but no clinical parameters such as tooth mobility were measured. Healing outcomes in the Ledermix group after 60 min of dry time were 59% favorable and in the Ca(OH)_2_ group 14% (*p* = 0.004). However, the absence of splinting and the temporary administration of a soft diet deviate from recommended protocols, and key methodological aspects remain unclear, including the distribution of interventions among the animals and tooth maturity. Moreover, no untreated control group was included. One clinical study by Day et al. (2012) investigated replanted avulsed teeth in children in a randomized controlled trial, in which half of the teeth were treated with Ledermix and the other half with calcium hydroxide. The authors reported that ankylosis occurred in unsuccessful cases regardless of the intracanal medicament used.

In conclusion, this in‐vitro model challenges the understanding that calcium hydroxide diffusion through dentinal tubules alone can be expected to change the periradicular pH in a clinically relevant way, at least in an in vitro setting. Due to model limitations, no direct clinical inference can be drawn, but the results suggest that clinical guidelines recommending against the use of calcium hydroxide during the first 2 weeks after trauma may need to be reconsidered. Future studies should quantify pH changes in relation to apex diameter and integrate biological parameters such as buffering systems, with the ultimate goal of gaining insight into whether calcium hydroxide can or cannot be used immediately after dislocation injuries.

## 5 Conclusion

This in vitro study showed no alterations of the extraradicular pH for both calcium hydroxide and Ledermix root canal dressings in a sealed‐apex model. Also, the presence of cemental defects was not associated with external alkalinisation. Only in teeth with unsealed apices, the calcium hydroxide dressing led to an increase of the pH.

Within the limitations of this experimental model, these findings suggest that dentinal diffusion of intracanal medication alone may have a limited effect on the external root surface pH after dental trauma. Given the in vitro study design, these findings should not be translated to clinical decision‐making. Further studies are required to define the clinical effect for different root canal dressings after dental trauma.

## Author Contributions

Alina Paganini, Eva Magni, and Andreas Filippi conceived and designed the study. Anne Géraldine Guex helped in experimental design and provided lab infrastructure. Alina Paganini and Eva Magni performed all experimental evaluations. Wadim Leontiev conducted the statistical analysis. Alina Paganini, Eva Magni, and Wadim Leontiev were responsible for the analysis, interpretation of data, and writing of the manuscript. Thomas Connert, Anne Géraldine Guex, and Andreas Filippi revised the manuscript critically for intellectual content. The final version for publication was approved by all authors.

## Ethics Statement

Extracted human teeth used in this study were obtained based on clinical indications unrelated to the research. The use of the teeth was approved by the local research ethics committee (EKNZ UBE‐15/111).

## Consent

Written informed consent was obtained from all participants or their legal guardians for the use of extracted teeth for research purposes.

## Conflicts of Interest

The authors declare no conflicts of interest.

## Supporting information


Supporting File 1

Supporting File 2

## Data Availability

The dataset obtained from the study is securely stored in encrypted form at the University Center for Dental Medicine Basel UZB. Upon reasonable request to the corresponding author, the data supporting this article will be made available.
