# Supplementary material for: Effect of Root Canal Dressings on Extraradicular pH in a Sealed Apex Model: An In Vitro Study
Source: Clin Exp Dent Res. 2026 Sep 27;12(5):e70467. doi: 10.1002/cre2.70467 (PMC13616235; doi:10.1002/cre2.70467)
Supplement: Supplementary file 2 [file CRE2-12-e70467-s002.docx]

**PRILE 2021 Flowchart**

RATIONALE/JUSTIFICATION Current literature is inconclusive regarding initial use of calcium hydroxide after dental luxation injuries. The concern is about potential extraradicular pH elevation and additional cell damage.

AIM/HYPOTHESIS To quantify extraradicular pH changes resulting from the dentinal diffusion of calcium hydroxide and Ledermix in human teeth using a sealed-apex model.

ETHICAL APPROVAL (IF APPLICABLE) Extracted human teeth used in this study were obtained based on clinical indications unrelated to the research. The use of the teeth was approved by the local research ethics committee (EKNZ UBE-15/111).

SAMPLES 35 cryopreserved human single rooted premolars.

EXPERIMENTAL AND CONTROL GROUPS, INCLUDE INDEPENDENT VARIABLES Group 1 (n=5) Calcium hydroxide, w cemental defects, w apical seal Group 2 (n=5) Calcium hydroxide, w/o cemental defects, w apical seal Group 3 (n=2) Calcium hydroxide, w/o cemental defects, w/o apical seal Group 4 (n=5) Ledermix, w cemental defects, w apical seal Group 5 (n=5) Ledermix, w/o cemental defects, w apical seal Group 6 (n=2) Ledermix, w/o cemental defects, w/o apical seal Group 7 (n=5) Saliva, w cemental defects, w apical seal Group 8 (n=5) Saliva, w/o cemental defects, w apical seal Group 9 (n=1) Saliva, w/o cemental defects, w/o apical seal

OUTCOME(S) ASSESSED, INCLUDE DEPENDENT VARIABLES AND TYPE Teeth were stored in NaCl solution. External pH of the solution was measured on days 1, 3, 5, 7 and 14.

METHOD USED TO ASSESS THE OUTCOME (S) AND WHO ASSESSED THE OUTCOME(S) pH electrode was inserted in the NaCl solution to record the pH value. The measurements were randomly cross-checked by two investigators (A.P. and E.M.)

RESULTS Calcium hydroxide in teeth without apical sealing caused a sustained external alkalinisation (± pH 12). In teeth with apical sealing, no significant external rise in pH occurred for any dressing over time compared to day 1; only one sealed specimen with calcium hydroxide dressing reached pH 9.1 transiently. The pH in Ledermix and saliva groups remained roughly neutral irrespective of sealing or cemental defects

CONCLUSION(S ) In a sealed-apex model, intracanal dressings did not raise the external pH significantly by dentinal diffusion.

FUNDING DETAILS Center of Dental Traumatology, University Center for Dental Medicine Basel, University of Basel (grant number AZ 25-01 ZUZ).

CONFLICT OF INTEREST The authors confirm that they have no conflict of interest.

***From: Nagendrababu V, Murray PE, Ordinola-Zapata R, Peters OA, Rôças IN, Siqueira JF Jr, Priya E, Jayaraman J, Pulikkotil SJ, Camilleri J, Boutsioukis C, Rossi-Fedele G, Dummer PMH (2021) PRILE 2021 guidelines for reporting laboratory studies in Endodontology: a consensus-based development. *International Endodontic Journal* May 3. doi: 10.1111/iej.13542.** [**https://onlinelibrary.wiley.com/doi/abs/10.1111/iej.13542**](https://onlinelibrary.wiley.com/doi/abs/10.1111/iej.13542)**.**

**For further details visit: http://pride-endodonticguidelines.org/prile**
